# Implementing Artificial Intelligence in Traditional B2B Marketing Practices: An Activity Theory Perspective

**DOI:** 10.1007/s10796-022-10294-1

**Published:** 2022-05-26

**Authors:** Brendan James Keegan, Denis Dennehy, Peter Naudé

**Affiliations:** 1 School of Business, Maynooth University, Maynooth, Ireland; 2 School of Management, Swansea University, Wales, UK; 3 Manchester Metropolitan University, Manchester, UK

**Keywords:** Artificial Intelligence, Activity Theory, B2B Marketing

## Abstract

Anecdotal evidence suggests that artificial intelligence (AI) technologies are highly effective in digital marketing and rapidly growing in popularity in the context of business-to-business (B2B) marketing. Yet empirical research on AI-powered B2B marketing, and particularly on the socio-technical aspects of its use, is sparse. This study uses Activity Theory (AT) as a theoretical lens to examine AI-powered B2B marketing as a collective activity system, and to illuminate the contradictions that emerge when adopting and implementing AI into traditional B2B marketing practices. AT is appropriate in the context of this study, as it shows how contradictions act as a motor for change and lead to transformational changes, rather than viewing tensions as a threat to prematurely abandon the adoption and implementation of AI in B2B marketing. Based on eighteen interviews with industry and academic experts, the study identifies contradictions with which marketing researchers and practitioners must contend. We show that these contradictions can be culturally or politically challenging to confront, and even when resolved, can have both intended and unintended consequences.

## Introduction

The application of artificial intelligence (AI) in B2B marketing is receiving increasing interest from marketing scholars (Huang & Rust, 2021; Paschen et al., 2020a). Organisations are already harnessing the power of AI to identify novel strategic options in large swathes of customer data that would have been overlooked by the human analysts (Bag et al., 2021; Behera et al., 2021), as well as offering potentially lower operating costs (Davenport et al., 2020). While the adoption of AI applications in the context of business-to-consumer (B2C) marketing has received significant attention from the marketing research community (e.g., Liu, 2020; Dwivedi et al., 2021b; Upadhyay et al., 2021) there is a noticeable absence of rigorous research that focuses on how AI applications can be used in the context of B2B marketing. As Kotler and Keller (2012, p. 182) assert that “more dollars and items change hands in sales to business buyers than to consumers” indicating that B2B marketing represents a larger proportion of industry compared to the B2C sector. Many studies have considered the influence of technological enhancements to B2B processes throughout the years (e.g., Jaakkola & Hakanen, 2013) but only a limited stream has delved into the realm of AI (Han et al., 2021). What studies do exist, tend to focus largely on understanding the procedural enhancements for firms (Leone et al., 2020; Paschen et al., 2020b) enhancing customer service experiences (Davenport et al., 2020), customer segmentation and profiling (Dwivedi et al., 2021a), and lead identification and scoring (De Bruyn et al., 2020).

The application of AI in B2B marketing brings increased complexity to both firms and their employees (Han et al., 2021). For example, De Bruyn et al. (2020) identify two key challenges from AI applications in marketing, namely, technological implementation, and accountability of automation. From a technological perspective, poor experiences with use of AI solutions purchased in B2B exchanges (e.g., chatbots) has led to harsh criticism about AI in this context (Castillo et al., 2020). In terms of accountability, Syam and Sharma (2018) suggest that AI is not yet fit for managing the complexities that are inherent in the B2B buying process, as it is a role that is heavily reliant on human intervention. AI in B2B is claimed to be exceptional at utilising past events to predict future trends (Davenport et al., 2020), but unable to adapt to changes in business scenarios (Dwivedi et al., 2021a). The accountability of AI revolves around reliance on pre-determined algorithms written by technology providers that are unable to perform their intended usage, such as facial recognition algorithms which have been accused of sexism and racism (Zou & Schiebinger, 2018). Trust in AI marketing solutions on the part of both end-users and B2B marketers is only recently being explored (e.g., Balakrishnan & Dwivedi, 2021). Hence, a deep understanding of the complications arising from AI adoption from the perspective of B2B marketing practitioners is critical. Furthermore, adoption of AI in B2B marketing presents challenges to the traditional view of B2B service ecosystems (Vargo et al., 2017) whereby third-party suppliers provide AI marketing solutions for marketing buyers resulting in the automation of marketing processes (Davenport et al., 2020). Whilst prior studies have examined issues regarding new technology within B2B networks (e.g., Jaakkola & Hakanen, 2013), the influence of AI adoption and implementation from the marketers’ perspective remains under studied (Borges et al., 2021; Chiu et al., 2021).

This study draws on Activity Theory (AT) to address this research gap. AT is pertinent to this study as it is rooted in practice (Schatzki, 1998) and it focuses on the relationships between material action, mind and society (White et al., 2016). Further, it serves as a suitable lens to understand the mediating role of artefacts (e.g., technology) and goal-directed human activity within its natural context (Cole & Engeström, 1993; Kaptelinin, 1996).

Contradictions, also referred to as ‘growth buds’ (Foot, 2001) are a fundamental concept in AT, and are historically accumulating structural tensions that occur within an activity and/or between multiple interrelated activities (Engeström, 2001; Karanasios, 2018). Contradictions manifest themselves as errors, problems, ruptures of communication, breakdowns, and clashes which can interrupt the fluent flow of work (Helle, 2000; Kuutti, 1995). Contradictions are viewed in AT as the motor of change (Allen et al., 2013) as they are a source of learning that, if addressed, can become the driving force for expansive learning and change in an activity system (Engeström et al., 1999; Hasan & Banna, 2012; Karanasios, 2018). In the context of this study, B2B marketing involves multiple actors (e.g., buyers, suppliers) whose work activities are increasingly being mediated by technologies such as AI, making them highly vulnerable to contradictions.

AT has inspired several theoretical reflections on the adoption and implementation of technologies, including information systems (Allen et al., 2013; Hasan et al., 2017; Malaurent & Karanasios, 2020; Effah & Adam, 2021), mobile technologies (Karanasios & Allen, 2014; Kietzmann, 2008; Ryu et al., 2005), and intermodal mobility ecosystems (Schulz et al., 2020a). AT has been used to examine co-creation (Schulz et al., 2020b), software development (Dennehy & Conboy, 2019; Dennehy et al., 2020), entrepreneurship (Jones & Holt, 2008), organisational learning (Engeström & Kerosuo, 2007), work redesign (Engeström, 2000), and human behaviour (White et al., 2016). It is through the lens of AT that study aims to answer the following question:

*What contradictions emerge through the adoption and use of AI in the context of B2B marketing practice?*

This study makes two important contributions. The first is a new understanding of AI adoption and use by marketers in the B2B domain, which extends beyond technical and procedural challenges. It shows that despite AI shaping marketing strategies, processes, and practices, significant implications for practice lie in the contradictions between perception and expectations of technological performance. The second is a theoretical contribution, through the application of AT that conceptualises AI in the context of B2B marketing. In doing so, it allows for the analysis of socio-cultural aspects of AI adoption and use, providing insights into the roles and cultural norms at play amongst B2B marketers and AI marketing solution suppliers. Through the concept of contradictions, AT is particularly salient in exposing the significant gaps in knowledge relating to marketing practice. This study also contributes by illuminating the contradictions between AI capabilities and traditional marketing practices, thus underpinning the transformative power of technological change and the challenges that emerge during its initial assimilation.

The paper is structured as follows. A background to AI-powered B2B marketing and AT is presented. Next, the research method, and the data collection and analysis techniques used are discussed. Then results of the research are presented, followed by a discussion, limitations, and directions for future research. The paper ends with a conclusion.

## Theoretical background

### AI-powered marketing

Industrial marketing is enjoying a boom in terms of the adoption of AI and machine learning (Bag et al., 2021). Large tech firms such as Google, Amazon and Microsoft offer applications that are highly effective in harnessing data from customers and other business stakeholders to generate insights for strategic decision-making (Davenport et al., 2020). Leone et al. (2020) suggest that AI is critical to modern industrial marketing, as it commands a role in pricing, buyer behavior (Martínez-López & Casillas, 2013), and sales (Syam & Sharma, 2018). Whilst technology advancements that increase efficiencies in marketing are not new, AI is unique in substituting high-level managerial actions, traditionally reliant on experienced marketers, with automated processes (Paschen et al., 2020a). Such enhancement of marketing through AI applications is a significant theme in most academic work to date (e.g., Bag et al., 2021; Davenport et al., 2020; Leone et al., 2020), at the expense of understanding further the implications for marketers. Hence, the limited thread of work on the interpretation of value created by AI marketing solutions represents a unique opportunity to examine the interplay between human agency and the capability of technology (Paschen et al., 2020a). The prevalence of studies on procedural enhancement is exemplified by Huang and Rust’s (2021) useful framework for adoption of AI in marketing by focusing on the enhancement of three processes, namely: marketing action (e.g., personalization), marketing research (e.g., segmentation), and marketing strategy (e.g., market analysis). Similarly, Paschen et al. (2020a) highlight how AI technology supports, and in some cases performs, traditional functions of the sales process such as lead generation and qualification, lead nurturing, lead scoring, developing competitor intelligence, and post-order customer service. At the same time, intriguing gaps emerge amidst the discussion of process enhancements, in terms of the perceptions and expectations of the marketing managers utilizing AI.

In contrast to the lion’s share of AI studies that present their discussions of the procedural enhancements achieved in an overtly positive light, the impact of the destruction of processes receives little attention. Authors have certainly outlined pitfalls with implementation of AI (e.g., De Bruyn et al., 2020), however even these commentaries are still reliant on critiques of the AI systems themselves, such as algorithmic coding bias. Within these application flaws, the literature also highlights the potential areas where frustrations amongst buyers and suppliers may emerge, leading to conflict within the buyer-supplier relationships (Dwivedi et al., 2021a). In their attempts to secure contracts, providers of AI marketing solutions are required to present a perception of a highly sophisticated and efficient system of AI and machine learning processes (Syam & Sharma, 2018). In doing so, the message being portrayed is that AI will do the job of the marketer better or will help the marketer do their job better (Paschen et al., 2020b), which has the potential to generate conflict within buyer-supplier relationships (De Bruyn et al., 2020). Equally, buyers of AI marketing solutions are presented with a unique proposition of having aspects their role being outsourced to an automated system (Davenport et al., 2020), and this may also create ripples of conflict across service ecosystems.

### The Evolution of Activity Theory

**Fig. 1.**
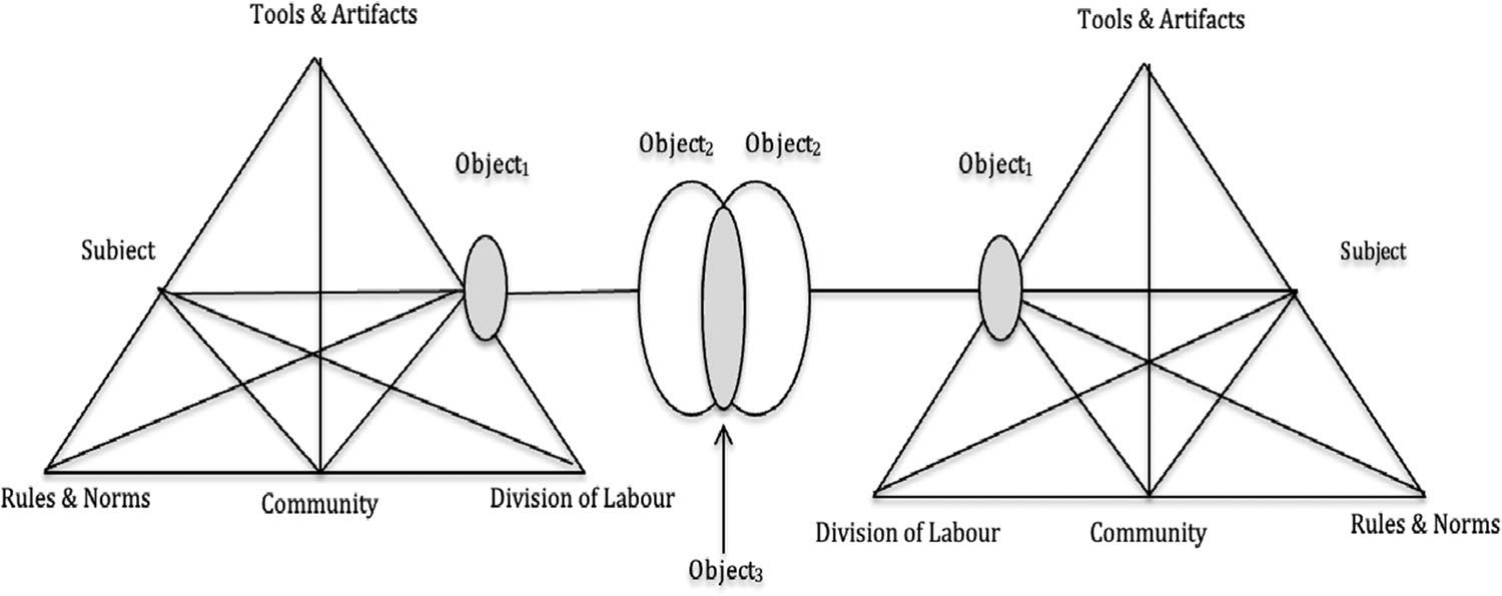
Interacting activity systems (Engeström et al., 1999)

AT is broadly defined as “a philosophical and cross-disciplinary framework for studying different forms of human practices as development processes, both individual and social levels interlinked at the same time” (Kuutti, 1996, p. 7). Rooted in Russian psychology, the first generation of AT was developed by Vygotsky (1978) in the 1920s and 30s and focused on the notion of ‘mediation’ (Engeström, 2001). The second generation of AT was proposed by Leontiev (1981) who argued that a limitation of Vygotsky’s work was that the unit of analysis was the ‘individual’ rather than ‘collective activity’ (Engeström, 2001). Third-generation AT emerged from the seminal work of Engeström (1987) who extends the core elements of an activity system (e.g. mediating artifacts, subject, and object) by acknowledging the wider context of the activity, namely rules and norms, community, and division of labour (see Fig. 1), as well as a minimum of two interacting activity systems (Allen et al., 2013). The notion of interlinked systems makes AT particularly well-suited to the analysis of complex collaborative work (Irnazarow et al., 2019), for example, the multiple teams or organisations involved in B2B marketing. At the core of third-generation AT is the notion that a subject (a person or collective) is driven by a motivation to act upon an object (a person, collective, or thing) using cultural-historical tools which include technologies, mental tools, and language (Karanasios & Allen, 2014).

Engeström (2001) proposes that the object moves from an initial *‘unreflected’* state (Object_1_) to a collectively meaningful object (Object_2_) constructed by the activity system to a potentially shared or co-constructed object (Object_3_). The six elements of the activity system (Kuutti, 1995; Engeström, 2000) are described as: (i) ‘Tools and artifacts’ which mediate the relationship between subject and object, (ii) ‘subject’ refers to the person engaged in an activity and who acts according to their own motives and goals, (iii) ‘object’ refers to the raw material or problem space which is transformed into outcomes with the aid of physical or symbolic, external and internal tools, (iv) ‘rules and norms’ are the explicit and implicit regulations, norms and conventions that constrain actions and interactions within the activity system, (v) ‘community’ comprised of multiple individuals and /or sub-groups who share the same general object in which the subject is performing the activity, and (vi) ‘division of labour’ refers to both the horizontal division of tasks between members of the community and the vertical division of power and status.

The five principles of AT and their relevance in this study are listed in Table 1. It is not compulsory to explicitly study the presence of all five principles in an AT oriented study (cf. Allen et al., 2013; Allen et al., 2014; Chen et al., 2013; Karanasios & Allen, 2014; Kuutti, 1995; Nardi, 1996).

**Table 1:** Principles of AT

Principle	Description (Engeström, 2001, p. 137)	Relevance to this study
1	*Collective, artifact-mediated and object-mediated:* The activity system is seen in its network of relationships to other activity systems, is taken as the prime unit of analysis.	B2B marketing is a collective, AI-mediated activity system and is the unit of analysis.
2	*Multi-voicedness:* An activity system is always a community of multiple points of view, traditions, and interests. The division of labour in an activity creates different positions for the participants, the participants carry their own diverse histories, and the activity system itself carries multiple layers and strands of history engraved in its artifacts, rules, and conventions. The multi-voicedness is multiplied in networks of interacting activity systems. It is a source of innovation, demanding actions of translation and negotiation.	To capture the “multi-voicedness” of the interacting B2B activity systems, interviews were conducted with subjects and community members of the activity systems.
3	*Historicity:* Activity systems take shape and get transformed over lengthy periods of time. Their problems and potentials can only be understood against their own history. History itself needs to be studied as local history of the activity and its objects, and as history of the theoretical ideas and tools that have shaped the activity.	The historical context of the B2B organisations include changes to organisational processes, structures, culture, and human resources over a period.
4	*Contradictions*: Are a source of change and development and are not the same as problems or conflicts. Contradictions are historically accumulating structural tensions within and between activity systems that generate disturbances and conflicts, but also innovative attempts to change the activity.	Contradictions that manifested due to misaligned understanding within and between the B2B activity systems that were catalysts for change within and between the B2B activity systems.
5	*Expansive transformation:* Activity systems move through relatively long cycles of qualitative transformations. As the contradictions of an activity system are aggravated, some individual participants begin to question and deviate from its established norms. In some cases, this escalates into collaborative envisioning and a deliberate collective change effort.	Transformational changes to the ways people conduct their marketing activities due to the successful adoption, adaptation, and assimilation of AI technology in B2B marketing.

Contradictions “refer to anything within the system that opposes the overall motive of the system, the aim or purpose that subjects within the system are individually or collectively striving toward” (Allen et al., 2013, p. 840). Contradictions expose the dynamics and inefficiencies within activity systems, as well opportunities for change (Helle, 2000) that can shape the activity (Engeström, 2001; Wiredu & Sørensen, 2006).

Within an activity system, contradictions are a cultural-historical force that lead to activities that are continuously evolving and transforming (Allen et al., 2013) in which “equilibrium is an exception and tensions, disturbances and local innovations are the rule of thumb and the engine of change” (Cole & Engeström, 1993, p. 8). Contradictions may not be obvious, openly discussed, or be culturally or politically challenging to confront (Allen et al., 2013; Capper & Williams, 2004). A related problem is that contradictions are not always acknowledged or resolved (Murphy & Manzanares, 2008). Contradictions can be resolved through tool-mediated change which may result in several levels of congruency that ultimately have a positive influence on the system (Karanasios & Allen, 2014; Mursu et al., 2007). These congruencies can be immediate where things or activities work better in some way or potential areas for longer term congruencies (Allen et al., 2014). Engestrom (1987) proposes four levels of contradictions that can occur in an activity system: (1) Primary contradictions which occur inside an element, (2) Secondary contradictions occur between two elements, (3) Tertiary contradictions describe potential problems caused by the relationship between an existing activity system and its more evolved object or outcome, and (4) Quaternary contradictions occur when there is conflict between interacting activity systems.

Tools are emphasised in AT as they mediate human activity and their influence on either the object or subject (Hasan et al., 1998, 2010). Tools can be physical (i.e., artifacts, technologies, mobile devices) which are used directly in production and produce changes in the object, or psychological (i.e., language, signs, models, cultural systems, virtual realities) (Hasan et al. 2010; White et al., 2016).

### AI-based B2B marketing as an interacting activity system

**Fig. 2:**
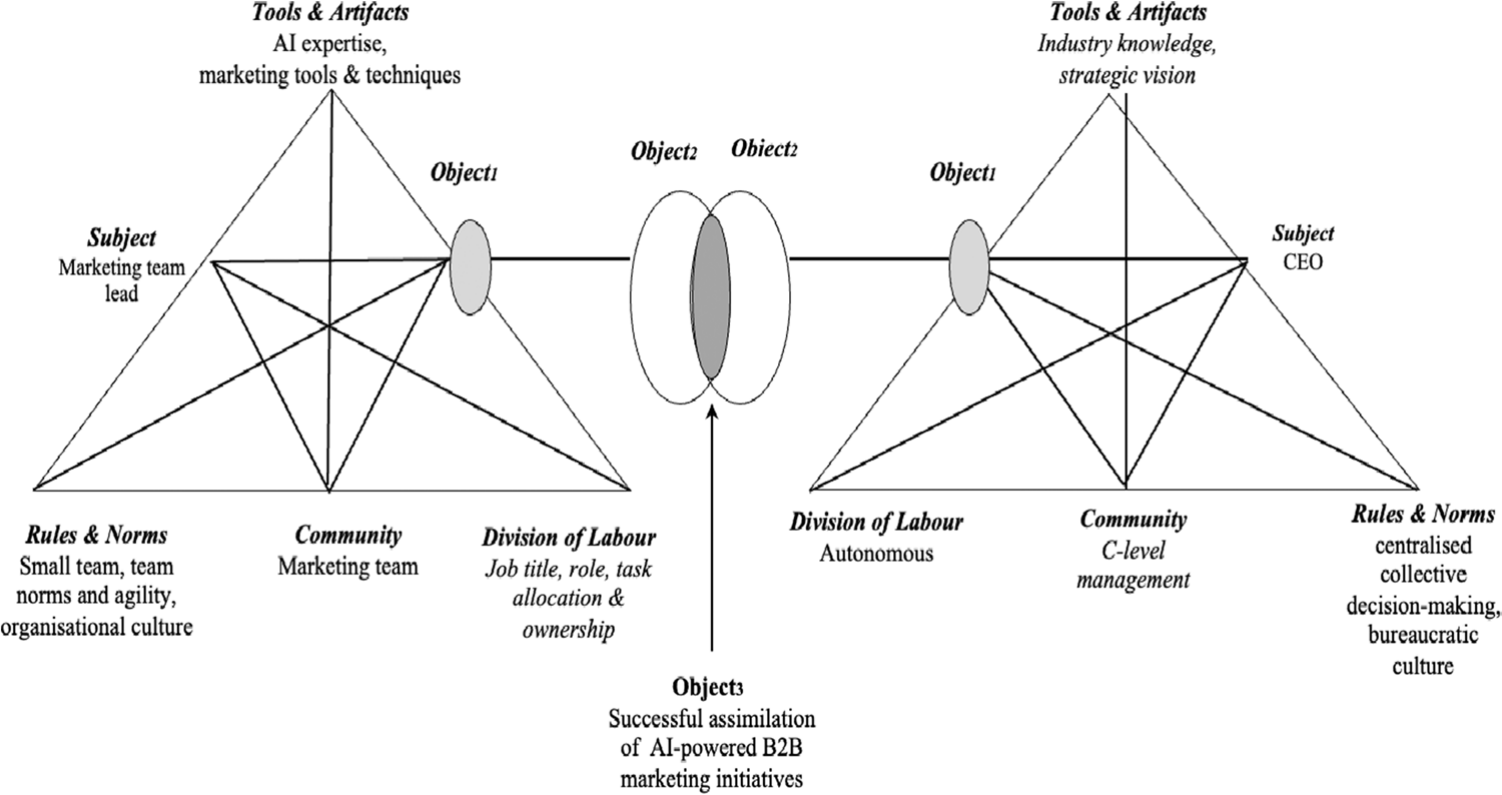
Instantiation of interacting AI-marketing activity systems

An instantiation of the AI B2B marketing activity system in this study is presented in Fig. 2 which illustrates the collective activities, by using the concept of shared objects. The activity system on the left is that of the AI solution providers that includes the subject (marketing manager), who manages the community (data scientists, technologists, and designers). Interactions between the subject and community, as well as within the community itself, are influenced by “rules and norms” (organisational policies and culture). These rules and norms determine the degree of shared understanding and commitment of the expected outputs (i.e., AI-powered B2B marketing) of the community. AI technologies mediate and support this activity. Coordination of activities within the AI-powered B2B marketing activity system is influenced by “division of labour” (job title and associated responsibilities) between members of the community and the subject (marketing manager). The AI-powered B2B marketing activity system is further influenced by interactions with other activity systems external to the organisation, which is the “client” (e.g., potential purchaser of the AI solution). This activity system is represented on the right side of Fig. 2 and it consists of a management team (community) that is responsible for the strategic positioning of the company in B2B marketing domain. Knowledge of the industry and strategy mediate the work of this community, who report to the CEO (subject). Interactions between the subject and community, as well as within the community itself, are influenced by these “rules and norms” (organisational policies and culture) of the activity system.

Due to the novelty of AI in B2B marketing and limited knowledge of its use in B2B marketing, members of both activity systems have their own ‘un-reflected’ (Object_1_) understanding of what AI-powered marketing is about and how best to deploy it. As the adoption of AI technology progresses, a collective understanding of how to adapt and implement AI (Object_2_) is constructed by the interacting activity systems, followed by the successful assimilation of AI-powered B2B marketing via a sense of shared understanding and ownership (Object_3_) by members of both interacting activity systems (cf. Engeström, 2001). While this study focuses on a specific instantiation of AI-powered in the context of B2B marketing, its conceptualisation can be generalised to study other digital marketing ecosystems such as B2C.

## Research method

The study sought to identify individuals who could offer the most cogent interpretation of AI adoption in B2B marketing. As a result, eighteen domain experts were selected using role-based sampling (Noy, 2008) as they had extensive academic and industry experience and knowledge of AI and innovation management, and are listed in Table 2. Participants varied in terms of their role, industry experience and use of digital marketing design innovations. All participants were contacted through a notification on a UK digital industries trade association newsletter, resulting in eight responses. A snowball effect followed whereby introductions were made by the initial participants throughout their networks. Additionally, academic researchers were approached who were actively researching AI in B2B marketing or advising on policy decisions relating to AI in business. These viewpoints added an objective perspective of AI in practice.

**Table 2:** Interviewee profile

**Code**	**Job role**	**Actor role in B2B ecosystem**	**Domain of expertise**	**Years of experience**	**Years at current organisation**
P1	Director of Analytics and Insights	Buyer	Telecoms	20	5
P2	Director of Research	Buyer	Telecoms	20	10
P3	Head of Technological Procurement	Buyer	Financial Services	20	5
P4	Head of Cloud Engineering	Buyer	Financial Services	16	3
P5	R&D Project Manager	Buyer	Pharmaceutical	15	15
P6	R&D Director	Buyer	Healthcare	13	13
P7	AIML Marketing Solutions Provider	Supplier	Healthcare	8	4
P8	Information Architect	Buyer	IT	13	4
P9	AIML Marketing Solutions Provider	Supplier	SAAS	10	4
P10	Head of Marketing	Buyer	eCommerce/Retail	12	10
P11	AI Marketing Solutions Provider	Supplier	eCommerce/Retail	10	8
P12	AI Marketing Solutions Provider	Supplier	eCommerce/Retail	10	3
P13	AI Marketing Solutions Provider	Supplier	SAAS	10	5
P14	Academic Expert in AI	Researcher	Computer Engineering	10	10
P15	Academic Expert in AI	Researcher	Computer Engineering	15	15
P16	Academic Expert in Marketing	Researcher	Marketing	12	12
P17	Academic Expert in Marketing	Researcher	Marketing	10	10
P18	Academic Expert in Marketing	Researcher	Marketing	8	8

To improve reliability and repeatability, a ‘clear chain of evidence’ was created from data collection through to drawing of conclusions. Data were collected over the period of March to May 2020, against a backdrop of a national UK lockdown due to Covid-19, which proved effective in recruitment of online interviews. As a result, semi-structured interviews were conducted online with the domain marketing experts, of which thirteen were practitioners, and five academic researchers who were actively involved in industry-oriented AI projects. An interview guide was developed to enable a significant level of subsequent probing on each point. Interviews lasted between 40 and 55 minutes. Interviews were recorded using Zoom whilst simultaneously transcribed using Otter, an AI-based transcription software, proof-read and annotated.

Data was then analysed using the elements of AT (e.g., Schulz et al., 2020b), as conceptual frameworks enable the researcher to classify the collected data into ‘intellectual bins’ and it facilitates the theorising process (Miles & Huberman, 1984). Data analysis was guided using open (i.e., categorising data) and axial coding (reconstructing the data in new ways after open coding) as proposed by Strauss and Corbin (1998). Two researchers coded the interview data to identify emerging novel themes and axial codes (see Table 3). The third author reviewed the codes independently, to ensure intercoder reliability was established.

**Table 3:** Sample of codes used in the analysis

**Sample Quote**	***Open Code***	***Axial Code***
*Many people felt that we didn't get a return on investment on the AI as it didn't do anything for us. But this was mainly because we were trying to fix the wrong problem. What people were really looking for was something to help them make predictions and scenario planning such as What if? What if we did this? What would be likely to happen? Or where might we go into something with diminishing returns?* (P9)	Object	Emerging factors/shared understanding (e.g., Object_3_)
*We need to find the right balance between the use of AI and the right level of human wisdom overlaid on top to ask questions such as, What does that mean based on what's happening in the market? What does that mean based on our brand? Based on our capability to execute those different channels equally.* (P12)	Tools & Artifacts	Emerging factors/assimilation of AI

## Results

In this section we use the elements of AT to illuminate the most prominent contradictions associated with each element in the B2B activity system. Identifying these contradictions captures the *multi-voicedness* of the collective nature of the interacting B2B activity systems. Next, by using the concept of contradictions, we illuminate their manifestations within the broader social context when AI is being implemented into the interacting B2B marketing activity systems. It is within this context of B2B marketing that we focus our analysis on identifying the contradictions in the adoption of AI-powered technologies and understanding how these contradictions influence the implementation of AI. Table 4 lists the key problems that emerged from the analysis and are categorised based on the elements of AT and the associated contradiction.

**Table 4:** Manifestations of contradictions at the level of the activity system

AT elements	B2B activity system	Data source	Contradiction type
Shared object	*Supplier*	*I think where probably the best examples would be around the business challenges like optimising marketing spend, which in consumer businesses often focuses a lot on optimising the marketing mix and using attribution to build to essentially predict where we should spend. And as a result, people feel we didn't get a return on investment, or the AI didn't do anything for us. (P6)*	*Primary*
Tools	*Supplier*	*There are problems related with the data, such as to what extent your data is representative of all customer groups you have. The danger there is that you are making assumptions and you are building a predictive model based on one type of customer and it doesn't work for the other type of customer. So, the challenge here is the quality and the representativeness of the data. (P3)*	*Secondary*
Community	*Supplier*	*We try to minimise these challenges but a classic story that I hear, which is not new is that there's been a large investment made in building something and then the organizations never got any value from it. This is a new challenge. It might have been described around a data mining and modeling project 10 years ago, and now it's described as an AI project, the business challenges changed. (P7)*	*Tertiary*
Division of labour	*Supplier*	*You would have computer scientists use machine learning, and statisticians would use mathematics. To achieve that, what we've now seen, perhaps with the convergence of some of those roles, and some of those skill sets are co-working much closer together. There's also quite a tight relationship between how they're deployed and how they are designed. That either means you have slightly more DevOps style teams emerge, particularly with the technologies that are involved, or new roles could emerge in terms of making sure these things work well. (P4)*	*Tertiary*
Subject	*Buyer*	*There are challenges with the actual code of articulating the problem you're trying to fix, making sure you've got the right kind of business measures and feedback loops to make sure that models can work in practice. For example, in the live production scenario where you've got to deliver content in real-time to users, and deal with all those challenges. (P12)*	*Quaternary*
Rules & norms	*Supplier*	*A lot of businesses have set up either new data science teams, or they've extended existing analytics teams to include new roles which suggests the notion of having teams that sit within an innovation space within the It’s a recognition that maybe challenges the status quo as the idea that innovation team gives them license to be able to go explore doing things differently across the business, rather than having it kind of submerged into a particular department. (P2)*	*Quaternary*

Following Engeström’s (1987) four types of contradictions, we present an aggregated theme for each of the four types of contradictions that manifested in the context of AI-powered B2B marketing activity system (see Table 5).

**Table 5:** Four levels of contradictions in AI-powered B2B marketing

Types of Contradiction	Context of AI B2B marketing activity systems	Supporting data
Level 1: Primary contradiction	Misaligned value systems between the AI B2B solutions provider and client due to no shared understanding (e.g., Object_1_) of how AI could be used to drive B2B marketing in the context of the client’s business.	*I think the best examples would be around the business challenges like optimising marketing spend, which in consumer businesses often focuses a lot on optimising the marketing mix and using attribution to build to essentially predict where we should spend. And as a result, people feel we didn't get a return on investment, or the AI didn't do anything for us. (P6)*
Level 2: Secondary contradiction	Management teams not taking into consideration the disruption to current work practices as marketing team assimilate AI technology into existing work processes.	*There are problems related with the data, such as to what extent your data is representative of all customer groups you have. The danger there is that you are making assumptions and you are building a predictive model based on one type of customer and it doesn't work for the other type of customer. So, the challenge here is the quality and the representativeness of the data. (P3)*
Level 3: Tertiary contradiction	Misalignment of expected and actual benefits of AI technology within the community (e.g., C-level management) of the AI-solutions client, in terms of their daily work practices and the nature of their work.	*We try to minimise these challenges but a classic story that I hear, which is not new is that there's been a large investment made in building something and then the organizations never got any value from it. This is a new challenge. It might have been described around a data mining and modeling project 10 years ago, and now it's described as an AI project, the business challenges changed. (P7)*
		*You would have data scientists use machine learning, and statisticians would use mathematics. To achieve that, what we've now seen, perhaps with the convergence of some of those roles and skillsets are co-working much closer together. There's also a tight relationship between how they're designed and deployed which either means you have slightly more DevOps style teams emerge, particularly with the technologies that are involved, or new roles could emerge in terms of making sure these things work well (P4)*
Level 4: Quaternary contradiction	As the AI B2B solutions provider activity system interacts with the client’s activity system, the emergence of contradictions necessitates more change in both activity systems.	*There are challenges with articulating the problem you're trying to fix, making sure you've got the right kind of business measures and feedback loops to make sure that models can work in practice. For example, in the live production scenario where you've got to deliver content in real-time to users, and deal with all those challenges. (P12)*
		*A lot of businesses have set up either new data science teams, or they've extended existing analytics teams to include new roles which suggests the notion of having teams that sit within an innovation space within the business. It’s a recognition that maybe challenges the status quo as the idea that innovation team gives them license to be able to go explore doing things differently across the business, rather than having it kind of submerged into a particular department. (P2)*

Exemplars of the manifestation of contradictions in AI-powered B2B marketing is provided in the remainder of this section. Analysis illustrated key examples of how both individual and collective motivations of stakeholders represented conflicting viewpoints (e.g., Object_1_) that can either inhibit or lead to a shared, reflected understanding and commitment of AI-powered marketing (e.g., Object_3_). Findings pointed towards the notion that the utilisation of AI in B2B marketing provided beneficial enhancements to marketing practice, however the actualisation of that benefit varied amongst participants. On the one hand, the primary motivation for the B2B marketing team is to use AI to improve the efficiencies of the processes they perform.

*AI marketing solutions has really picked up and you've got the likes of AWS with Sagemaker taking away the complexities of building up AI models in a way that we can embed AI and machine learning into our existing application architecture without really having to get into a lot of the detail of developing the models themselves. One of the other things I work a lot on is intelligent decision making where AI and machine learning are using deep learning to really understand the many routes to get to a marketing decision. (P8)*

Interviewees indicated how successful adoption and implementation of AI in their respective organisations offered significant benefits in the ability to harness large volumes of customer data and automating processes and procedures. In effect, the perceived outcome of AI adoption is streamlining their roles as marketing managers. However, while senior management embraced the same benefits of such innovations to marketing practice, they were also motivated by cost reduction, division of labour amongst marketing teams and creating the external perception of technological prowess through innovation.

*Benefit number one is cost saving. Benefit number two is a genuine change in functionality that differentiates and makes the product more attractive and differentiated from rivals. Benefit number three, is perhaps the one that's you know, must be expressed a little bit more delicately, but it is the marketing opportunity, the brand positioning, the ability to be perceived to be at the front of technology and of development and always pushing the boundary. It comes down to those three things, in my opinion, excellent saving cost, promoting artificially or genuinely differentiating your value proposition. (P6)*

Contradictions between B2B managers and AI technologies emerged due to a perceived threat to their managerial role in the future (e.g., secondary and tertiary type of contradictions). It was suggested that senior management present a differing desire to automate processes to effectively replace the jobs that humans would do.

*If individuals are replaced by machine applications or automated, we still end up losing jobs. However, because AI is based on an algorithm which probably gave its designer a job, we are making companies more efficient, but losing their job is a worrying thing. Is AI going to provide new jobs for these people? (P17)*

Participant descriptions of AI adoption in marketing also presented a unique juxtaposition whereby B2B marketing managers were keen to outsource pivotal processes and procedures to third parties. Suppliers ranging from large tech firms who provide bespoke large scale marketing solutions, to smaller specialist firms offering niche solutions. As a result, the numerous AI marketing solutions providers create a cluttered marketplace with competitive firms jostling for position by offering newer and more effective innovations. Whilst outsourcing is commonplace within B2B marketing, it is a unique perspective that when contracted, the external third party commandeers a significant control over the marketing procedures and datasets of their client.

*It’s a cluttered market. A lot of third-party vendors use Amazon, Microsoft, with Azure labs. For example, they have a couple of sort of more retail oriented cross sell upsell style models, or recommendation type things. But again, there's a black box side to it too. You've got to measure them to make sure that they do and there's maybe a limited opportunity to be able to customise your business model. If you have a competitive market, you might have several businesses all attacking the same problem, then they are all trying to build the best model then you have a kind of subscription service or software as a service type thing where you're essentially renting usage of that algorithm. But it's still a slightly weird dependency. It's a bit like you've kind of subcontracted some key decision making to somebody that never tells you why they made the decision. But then the rates go up when you are dependent on a black box algorithm, on some IP that you don't own. (P7)*

Importantly, third party suppliers of AI marketing solutions exhibit a unique set of motivations in their attempts to do a good job for their clients, but also to restrict their access to the AI algorithms to protect their own intellectual property.

*There must be lots of black box algorithms in what we do. Its designed around creating value for the business, and we are renting that value to the customer who must be willing to put their faith in that black box. (P8)*

A similar view was shared by the Head of Technological Procurement,

*One of the big challenges is explainability. When you use machine learning, especially if you use unsupervised machine learning, you can't explain how the model came up with certain recommendations, and these can create problems if there are compliance issues. In the financial services, you need to prove that you're not discriminating against certain consumers. If you don't know what the model is deciding to offer to each customer, or how to calculate the interest rate etc. how can you explain it? There is an issue of compliance there. (P3)*

**Fig. 3:**
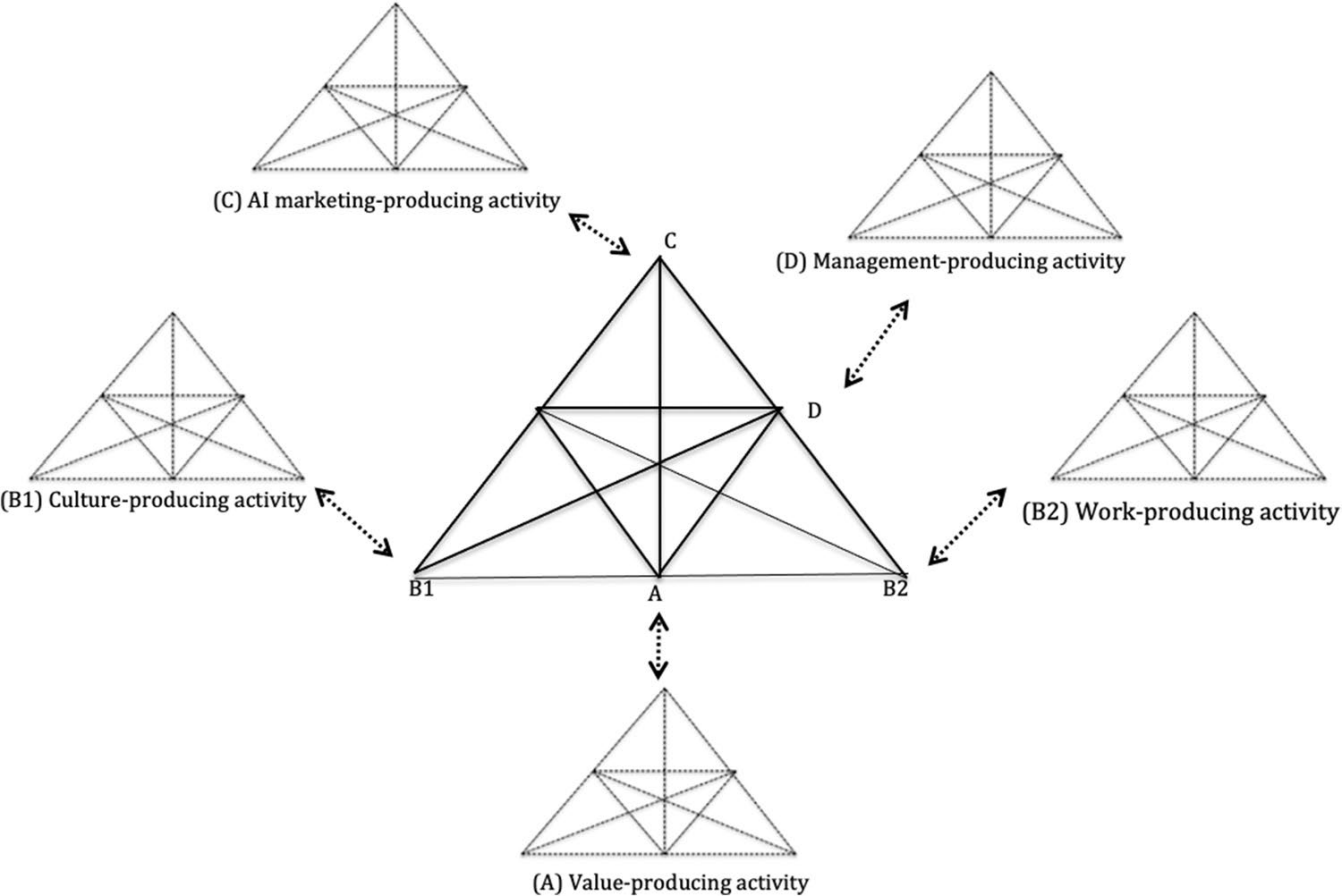
Inter-related contradictions

Through the process of distillation and deduction, we identify and categorise the type of contradictions that emerge during the implementation of AI-powered B2B marketing. Figure 3 illustrates these inter-related contradictions, namely:

- *Primary contradiction:* Misaligned value systems between marketing and management teams (A).
- *Secondary contradiction:* Conflicting rules, norms and roles between AI and current marketing practices (B1, B2).
- *Tertiary contradiction:* Disconnect between expected versus realised benefits of AI (C).
- *Quaternary contradiction:* Tensions between marketing and management activity system (D).

In summary, the findings provide novel insights relating to the successful implementation of AI-powered solutions into B2B marketing practices, whereby stakeholders are continuously learning, and expansive transformations occur at the level of the activity systems.

## Discussion, limitations, and future research

### Practical implications

Our study uses AT to represent AI-powered B2B marketing as a collective activity system that is highly vulnerable to conflict as object-oriented actions are “always, explicitly or implicitly, characterized by ambiguity, surprise, interpretation, sense making, and potential for change” (Engeström, 2001, p. 134). An implication for B2B management teams is the need to understand the contextual and cultural-historical influences of an existing B2B marketing activity system, and not to be consumed by the hype surrounding AI and other emerging technologies in general. Another implication for practice relates to the lack of trust, and fears of the actors in the AI B2B service ecosystem. A related implication is that the adoption and implementation of AI is not a binary process where B2B marketing teams simply accept this new way of doing work. Instead, successful implementation occurs over time in an evolutionary process, whereby management and marketing teams are continuously and iteratively learning, through the process of internalisation and externalisation (Leontiev, 1978), thereby ensuring that AI is embedded into the organisational culture, norms, and processes.

Finally, by using AT to conceptualise contradictions and opportunities for innovation, it provides managers with the opportunities to (i) understand the different perspectives that exist within and between the interacting activity systems, (ii) facilitate dialogue that will enable stakeholders to move from a state of no reflection (Object_1_) to a state of shared understanding and shared commitment (Object_3_).

### Theoretical implications

Drawing on contemporary literature, we frame the contributions of this study (cf. Corley & Gioia, 2011). The most salient theoretical contribution of this research is the use of AT as a theoretical lens to study the adoption and implementation of AI in the context of B2B marketing practices. In doing so, we use AT to conceptualise AI-powered B2B marketing as an evolving collective activity system, that is increasingly being mediated by digital technologies (Allen et al., 2011; Karanasios & Allen, 2014). Understanding the contextual and cultural-historical influences provides insight into the B2B marketing activity system, beyond the initial hype stage surrounding the adoption of AI technology. The findings reveal that the adoption and implementation of AI is not a binary activity, whereby B2B marketing teams simply accept this new way of doing work.

This study contributes to theory by addressing the lack of knowledge in industrial marketing research that specifically examines the impact of AI adoption from the marketer’s perspective (e.g., Davenport et al., 2020) and advancing understanding on the emerging contradictions. Much of the prior knowledge in the field tends to focus on technical aspects of AI applications in marketing (De Bruyn et al., 2020; Huang & Rust, 2021), overlooking socio-cultural dimensions, in particular the conflict and tensions that emerge within the service ecosystem, as well as within the data environment (Meadows et al., 2022). Moreover, our findings indicate how expectations of buyers tend to be high, as do the intentions of the suppliers of the AI services, whereas the actualisation of the AI service creates contrasting results. Such contradictions in terms of the expectations of AI is in stark divergence to the overtly positive perspective of the major works in this area that suggest that AI “will augment rather than replace human managers” (Davenport et al., 2020, p. 39). In this extension of the B2B marketing literature, this paper also advances understanding of the role of actors involved in the adoption and implementation of AI technology, which has received limited attention to date (De Bruyn et al., 2020; Leone et al., 2020). Specifically, through the identification of contradictions derived from the utilisation of AT, we theorise that the value systems between buyers and suppliers are misaligned, as well as in the upper echelons of management structures in B2B organisations. Therefore, by using AT as a lens to scrutinise AI adoption and implementation in the B2B domain, the findings advance understanding about the relationship between human agency, technological capabilities and associated challenges that emerge. Additionally, the study extends the generalizability of AT through its application and conceptual development of B2B marketing as a collective activity system, illuminating the associated contradictions that emerge.

Our theoretical contributions produce a clear implication for B2B marketing organisations aiming to advance their B2B marketing capabilities through the adoption of AI, highlighting the need to ensure ‘organisational readiness’, or else there is a high risk that the business value to be gained from the AI functionality will not be realised, and worse, the AI initiative could be prematurely abandoned. Furthermore, we identify how B2B marketing managers and business decision makers have a need to develop and implement internal support systems that can enable the marketing team to embed AI and digital technologies in general into the daily practices of the team and wider ecosystem, as this enables business value to be generated by such technologies.

Lastly, our study provides a methodological contribution that lies in the utilisation of AT to understand the phenomena of AI-powered B2B marketing. In doing so, the B2B marketing context serves to confirm the theoretical conceptualization as suggested by other AT works (Schulz et al., 2020a, 2020b). Hence, AT enabled us to illuminate contradictions between AI technologies and traditional B2B marketing practices, and the transformative power of contradictions, which can lead to changes within the B2B interacting activity systems.

### Limitations and future research

As with all research, there are limitations that present interesting directions for future research and are important when considering AI in the context of B2B marketing. First, the cultural-historical emergence of contradictions is inherently time bound. Therefore, other contradictions are likely to emerge as the B2B marketing ecosystem evolves and AI becomes embedded in the day-to-day activities of the marketing teams. Second, related to the previous limitation is that contradictions are not always visible or openly discussed, which means they may not always be resolved (Dennehy & Conboy, 2017; Dennehy et al., 2020). Third, as a qualitative interpretive study, the vast amount of data that is generated from AI-powered marketing was outside the scope of this study. Future research could apply advanced deep learning techniques (cf. Choudrie et al., 2021) to provide new understandings about sentiment of customers and other actors within the B2B marketing activity systems.

Future research could conduct a longitudinal study to examine the changing nature of contradictions and congruencies as B2B marketing teams become more confident with the use of AI and it becomes embedded within the B2B marketing activity systems. Future research could also focus on contextual factors such as the role of organizational and national culture as these have not been adequately explored in the context of B2B marketing and emerging technologies (Dwivedi et al., 2021b). Finally, future research could focus on the responsible and ethical design of (marketing) information systems (Dennehy et al., 2021) as a way towards digital transformation and sustainable societies (Pappas et al., 2018).

## Conclusion

This study was motivated by the need to address the gap in knowledge about the mediating role of AI in the context of B2B marketing - an inherently complex and socially embedded activity. The study illuminated contradictions that emerge during the adoption and implementation of AI in this context and how they can influence the successful implementation of AI in B2B marketing practices. It thereby contributes to the paucity in B2B marketing research that considers the socio-cultural influences of AI on marketing managers. In doing so, this study provides empirically-based insights surrounding the hype of AI in an area of B2B business practice, a largely neglected research domain. Finally, the results provide practical insights that can enable B2B practitioners to avoid pitfalls when considering the adoption and implementation of AI-powered technologies in the context of B2B marketing.
